# Association between the L55M and Q192R polymorphisms of the paraoxonase-1 gene and age-related macular degeneration: a meta-analysis

**DOI:** 10.5935/0004-2749.20210033

**Published:** 2021

**Authors:** Rebeca G. Elguezabal-Rodelo, Renata Ochoa-Precoma, Leonardo M. Porchia, Ricardo Perez-Fuentes, M. Elba Gonzalez-Mejia

**Affiliations:** 1 Facultad de Medicina, Benemérita Universidad Autónoma de Puebla, Puebla, México; 2 Laboratorio de Investigación en Fisiopatología de Enfermedades Crónicas, Centro de Investigación Biomédica de Oriente, Instituto Mexicano del Seguro Social, Delegación Puebla, Metepec, Puebla, México

**Keywords:** Ethnic groups, Macular degeneration, Polymorphism, genetic, Paraoxonase-1, Aryldialkylphosphatase, Grupos étnicos, Degeneração macular, Polimorfismo genético, Paraoxonase1, Arildialquilfosfatase

## Abstract

**Purpose:**

Paraoxonase-1 activity is associated with age-related macular degeneration. Two polymorphisms (L55M and Q192R) were shown to increase paraoxonase-1 activity and have been implicated in the development of age-related macular degeneration. The results of studies that have examined these polymorphisms are conflicting, showing no effect, as well as increased or decreased risk. Therefore, this meta-analysis was conducted to determine the effect of these polymorphisms on age-related macular degeneration.

**Methods:**

PubMed, EBSCO, LILACS, and Scopus databases, as well as and the retrieved bibliographies of publications were searched for case-control studies that examined for paraoxonase-1 polymorphisms and age-related macular degeneration. Data were analyzed using the Comprehensive Meta-Analysis Version 2.2 and the NCSS Statistical Version 2020 software. Genotype distributions were extracted and, depending on the level of heterogeneity, fixed effects or random effects models were used to calculate pooled odds ratios (ORs) with 95% confidence intervals (95% CIs) for the heterozygous, homozygous, dominant, recessive, and allelic genetic models.

**Results:**

Overall, for the L55M polymorphism, none of the genetic models demonstrated a significant association. However, for non-Asian populations, a significant association was determined for the heterozygous and dominant genetic models (OR_range_=1.24-1.27, p<0.05). For the Asian population, the heterozygous, dominant, and allelic genetic models demonstrated a benefit/protective factor (OR_range_=0.29-0.35, p<0.05). For the Q192R polymorphism, none of the genetic models demonstrated a significant association. However, when the cohort was grouped by ethnicity, a significant association was determined in the Asian population for the recessive and allelic genetic models (OR_range_=1.63-2.08, p<0.05). However, for the non-Asian population, there was no association observed. Also, there was no identifiable risk when the cohort was stratified into exudative and non-exu dative cases.

**Conclusions:**

The paraoxonase-1L55M po ly morphism increases the risk of developing age-related macular degeneration in non-Asian populations, whereas in Asian populations, the polymorphism exerts a protective effect. However, for the paraoxonase-1 Q192R polymorphism, only the Asian population demonstrated a risk of developing age-related macular degeneration.

## Abstract (pt)

**Objetivo:**

A atividade da paraoxonase1 está asso ciada à degeneração macular relacionada à idade. Dois polimorfismos (L55M e Q192R) mostraram aumentar a atividade da paraoxonase1 e foram implicados no desenvolvimento da degeneração macular relacionada à idade. Os estudos que exa minaram esses polimorfismos apresentaram resultados conflitantes: nenhum efeito, risco aumentado ou diminuído. Assim, esta meta-análise foi realizada para determinar o efeito desses polimorfismos na degeneração macular relacionada à idade.

**Métodos:**

Foi feita uma busca nos bancos de dados PubMed, EBSCO, LILACS e SCOPUS, bem como nas bibliografias compiladas das publicações, buscando-se estudos caso-controle que tivessem analisado os polimorfismos da paraoxonase1 e a degeneração macular relacionada à idade. Os dados foram analisados com *software* Comprehensive Meta-Analysis, versão 2.2, e NCSS Statistical, versão 2020. As distribuições de genótipos foram extraídas e, dependendo do nível de hete rogeneidade, modelos de efeitos fixos ou aleatórios foram utilizados para calcular razões de probabilidade (RPs) combinadas, com intervalos de confiança de 95% (IC 95%) para os modelos genéticos heterozigoto, homozigoto, dominante, recessivo e alélico.

**Resultados:**

Em geral, nenhum dos modelos genéticos demonstrou associação significativa para o polimorfismo L55M. Entretanto, em populações não asiáticas, foi determinada uma associação significativa para os modelos genéticos heterozigoto e dominante (RP_faixa_=1,24-1,27, p<0,05). Para a população asiática, os modelos heterozigoto, dominante e alélico mostraram um fator benéfico ou protetor (RP_faixa_=0,29-0,35, p<0,05). Para o polimorfismo Q192R, nenhum dos modelos genéticos demonstrou qualquer associação significativa. Porém, quando a coorte foi agrupada por etnia, determinou-se uma associação significativa na população asiática para os modelos genéticos recessivo e alélico (RP_faixa_=1,63-2,08, p<0,05). Contudo, nenhuma associação foi observada para a população não asiática. Não houve risco identificável quando a coorte foi estratificada em exsudativa e não exsudativa.

**Conclusões:**

Determinamos que o polimorfismo L55M da paraoxonase1 de fato aumenta o risco de desenvolvimento de degeneração macular relacionada à idade em populações não asiáticas, enquanto que em populações asiáticas, esse polimorfismo tem um efeito protetor. Porém, para o polimorfismo Q192R da paraoxonase1, apenas a população asiática demonstrou risco de desenvolver degeneração macular relacionada à idade.

## INTRODUCTION

Age-related macular degeneration (AMD) is the most common cause of legal blindness in industrialized countries^(1)^. AMD is the loss of sight in a specific region of the retina, which can present in numerous forms depending on the stage and type, such as choroidal neovascularization, retinal pigment epithelial abnormalities or detachment, disciform scar, geographic atrophy, and drusens^(2)^. However, the two most common forms are exudative (wet or choroidal neovascularization) and non-exudative (dry or geographic atrophy)^(3)^. The pathway for disease development is multifaceted and not fully understood. Evaluation of oxidative stress in AMD patients did demonstrate decreased paraoxonase-1 (PON1) activity when compared with the control group (132.27 ± 63.39 U/l vs. 312.13 ± 136.23 U/l respectively; p<0.001)^(4)^. However, according to Otocka-Kmiecik et al., multiple factors have to be considered while studying complex age-related diseases that have been associated with augmented paraoxonase activity^(5)^.

PON1 is a calcium-dependent esterase and lactonase produced in the liver. It is located on chromosome 7 q21.3-q22.1 and consists of nine exons. PON1 has antioxidant activity by hydrolyzing paraoxon, the active metabolite of organophosphates, such as parathion, diazinon, and chlorpyrifos^(6)^. Worldwide, the expression and activation of PON1 is highly variable^(3)^; however, it has also been shown to be affected by smoking and diet^(7,8)^. Smoking decreases PON1 activity by ~1.7-fold^(8)^, whereas diet can increase PON1 activity by 25-85%. However, PON1 polymorphisms have been shown to significantly affect its activity by 40-fold ^(7)^. To date, there are many polymorphisms identified, with the two most common being L55M and Q192R. The L55M polymorphism (rs854560, ClinVar variant ID: 13736) consists of a thymine to adenine change at nucleotide 163 in exon 3^(9)^. This results in a 0.25- and 0.58-fold decrease in activity for the heterozygous and homozygous genotypes, respectively, when the LL genotype is considered the wild type. Interestingly, for the LL genotype, obese individuals have higher enzymatic activity^(10)^. The Q192R polymorphism (rs662, ClinVar variant ID: 13735) consists of an adenine to guanine change at nucleotide 575 in exon 6^(9)^. Independent of the subject’s obesity, heterozygous and homozygous genotypes resulted in a 1.02- and 2.22-fold increase in activity, respectively^(10)^, when compared with the QQ genotype. Interestingly, for the Q192R polymorphism, this switch from glutamine to arginine also can change substrate specificity for certain substrates^(9)^.

Numerous studies have examined the association between these two PON1 polymorphisms and the development of AMD. For example, for the Q192R polymorphism, using the recessive genetic model, Ikeda et al. demonstrated an increased risk of developing AMD^(11)^, whereas Söğüt et al. demonstrated the opposite effect^(12)^. Since no consensus has been reached on the effect of PON1 polymorphisms on the development of AMD, we conducted this meta-analysis to determine whether the L55M or Q192R polymorphisms augment the risk of developing AMD.

## METHODS

### Study selection

PubMed, LILACS, Scopus, and EBSCO databases were searched until March 25, 2019 to identify the studies related to AMD and PON1 polymorphism. The following search terms or any derivations were used: PON1 or paraoxonase; AMD, macular, or eye; and SNP or polymorphism. Only publications in English were included.

### Inclusion and exclusion criteria

The included studies were selected based on the following criteria: case-control studies; information on genotype or allele distributions for each group studied; and clear definition of the compared populations. The exclusion criteria were: animal and *in vitro* studies; case reports; reviews; conference reports; and studies with incomplete data. The reference sections of retrieved publications were also reviewed for articles not identified by the electronic search.

### Data collection and study quality assessment

Separately, two investigators (RGER and ROP) collected the following data: name of the first author; year of publication; diagnostic criteria (laboratory and/or clinical); genotype distributions or allelic frequencies; genotyping method; and Hardy-Weinberg equilibrium (HWE). In case of discrepancies, a third investigator (MEGM) reviewed the publication and a consensus was reached. Study bias was assessed using the Newcastle-Ottawa scale. This scale evaluates three components: selection; comparability; and exposure. The possible scores ranged 0-9, with scores <4, 4-6, and ≥7 indicating low-, medium-, and high-quality studies, respectively.

### Statistical analysis

HWE was evaluated using the χ^2^ test, where a p-value >0.05 was considered in agreement. For each study, the crude odds ratio (OR) and 95% confidence interval (95% CI) were calculated. The Cochran Q-based χ^2^ test was used to assess the heterogeneity between the studies, and the inconsistently index (I^2^) was used to quantify the proportion of the total variation attributable to the heterogeneity between the studies. Using the NCSS Statistical Version 2020 software (NCSS, LLC.; Kaysville, UT, USA; ncss.com/software/ncss), we evaluated possible causes of heterogeneity by constructing Galbraith plots. When the Q-based χ^2^ test indicated a significant result (p<0.10) and the I^2^ was >50%, the random effects model was used. The pooled OR and the 95% CI were calculated using the random or fixed effects model. For the genotypic comparison, heterozygous (12 v 11), homozygous (22 v 11), dominant (12 + 22 v 11), recessive (22 v 12 + 11), and allelic (2 v 1) genetic models were applied. For the L55M polymorphism, the L allele is the wild type (1) and the M allele is the mutant (2). For the Q192R polymorphism, the Q allele is the wild type (1) and the R allele is the mutant (2). Each study was removed, one at a time, and the pooled OR was re calculated to evaluate the stability of the results. Moreover, we assessed the asymmetry of the funnel plot as well as used Begg-Mazumdar correlation test and Egger’s regression test to determine publication bias and small study effects. Statistical analysis was performed with the Comprehensive Meta-Analysis Version 2.2 (Biostat Inc., Englewood, NJ, USA).

## RESULTS

### Literature search and characteristics of the included studies

**Figure 1:**
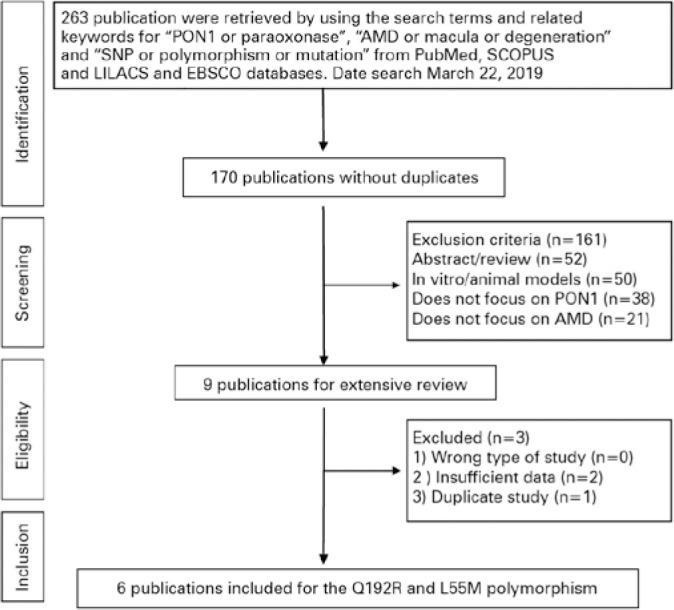
Flow chart of the literature review.

Following the removal of duplicates, we identified 170 publications using the search strategy (Figure 1). Of those, 161 publications were excluded for not being original research, not focusing on human subjects, not examining PON1, or including subjects that did not have AMD. Nine publications that investigated the association between L55M or Q192R polymorphism and AMD remained; however, one study was excluded because the data were used in a previous publication, and two were excluded for lack of sufficient data. Therefore, six publications were included in this meta-analysis, consisting of 1,420 cases and 978 controls in total.

AMD= age-related macular degeneration; PON1= paraoxonase-1.

Most of the studies included Caucasian populations: India^(13)^, Australia^(14)^, Ireland^(15)^, USA^(16)^, and Turkey^(12)^, whereas the last study was from Japan^(11)^. The characteristics of the studies are summarized in table 1. The most used genotyping method was polymerase chain reaction-restriction fragment length polymorphism. None of the studies presented significant study bias. Four studies were in HWE agreement and one study, in which the data were reported as allelic frequencies, indicated to be in agreement with HWE. However, for the study conducted by Pauer et al., the controls were not in agreement with HWE^(16)^.

**Table 1:** Characteristics of the included studies

Author, year	Country	AMD criteria	Group	L55M^a^	Q192R^a^	Age (years)	Male (%)	HWE^b^	NOS^c^
AnandBabu, 2016	India	AREDS	Cases	0/9/28	12/21/6	69 ± 1.3	64.6		8
			Control	1/7/19	8/16/2	53.2 ± 1.6	63.3	0.216	
Baird, 2004	Australia	International AMD classification system	Cases Control	20/30/12 46/51/18	35/22/4 59/44/10	70.4 ± 4.1 71.5 ± 6.5	46.8 45.2	0.053	8
Esfandiary, 2005^d^	Ireland	Angiographic assessment	Cases	60/188	49/188	76.6 ± N/I	38.2		7
			Control	65/190	57/190	78.8 ± N/I	50.0	N/A	
Ikeda, 2001	Japan	Choroidal neovascularization or vascularized pigment epithelial detachment	Cases Control	66/5/1 108/28/4	6/28/38 17/74/49	71.1 ± 8.1 70.6 ± 8.3	65.3 64.3	0.172	8
Pauer, 2010	USA	AREDS	Cases	352/441/126	458/437/64	77.0 ± N/I	60.1		8
			Control	161/164/43	155/146/69	76.3 ± N/I	N/I	0.001^*^	
Söğüt, 2013	Turkey	AREDS	Cases	76/60/6	22/79/41	66.6 ± 7.3	49.3		8
			Control	88/52/18	37/73/28	67.1 ± 8.2	52.1	0.462	

AMD= age-related macular degeneration; AREDS= Age-related Eye Disease Study; USA= United States of America; HWE= Hardy-Weinberg equilibrium; N/A= not applicable; N/I= not indicated; NOS= Newcastle-Ottawa Scale.

a = Values are the genotype distribution for the L55M polymorphism as LL (wild type), LM, and MM. For the Q192R polymorphism, the distribution is QQ (wild type), QR, and RR, respectively.

b = HWE agreement was determined using the χ^2^ test. P-values <0.05 were considered not in agreement with HWE and indicated with^*^.

c = NOS was used to determined study bias. Scores <6 denoted high-bias studies.

d = The results are the allelic frequency (wild type/mutant).

### PON1 L55M polymorphism increases the risk of developing AMD in non-Asian populations

**Figure 2:**
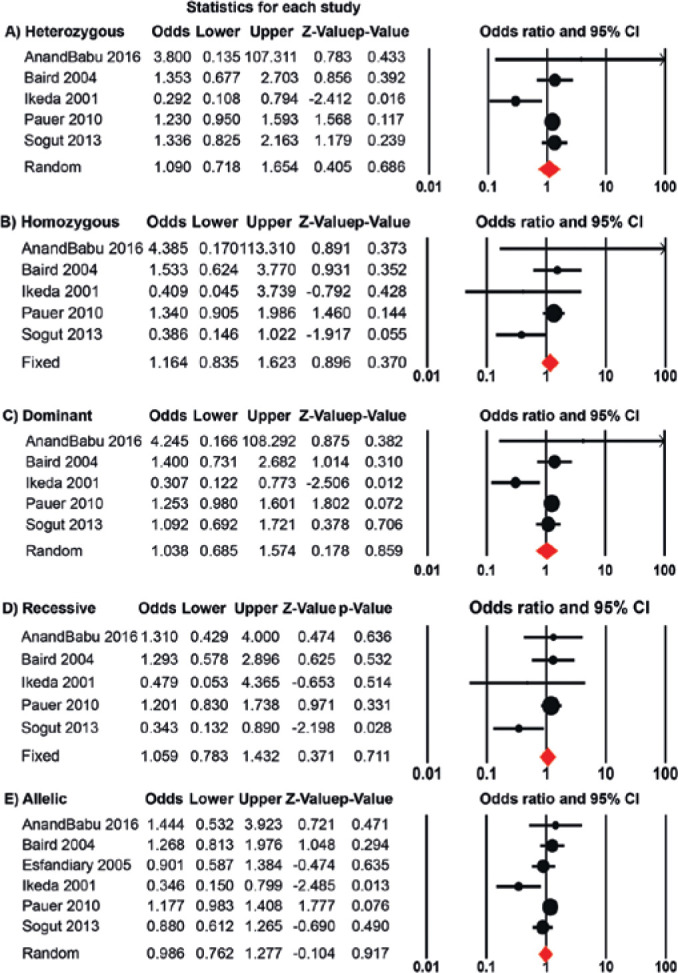
Forest plots to determine the risk of developing age-related macular degeneration (AMD) associated with the paraoxonase-1 (PON1) L55M polymorphism for the heterozygous (A), homozygous (B), dominant (C), recessive (D), and allelic (E) genetic models. The circles and horizontal lines correspond to the study-specific crude odds ratio (OR) and 95% confidence interval (95% CI), respectively. The area of the circles reflects the study-specific weight. The diamond represents the pooled OR and 95% CI, determined using either the fixed or random effects model, depending on the level of heterogeneity. Plots were generated using the Comprehensive Meta-Analysis software Version 2.2.

The heterozygous, dominant, and allelic genetic models presented significant heterogeneity and were analyzed using the random effects model. Among the five genetic models, none demonstrated a significant association between the L55M polymorphism and AMD (Figure 2). Interestingly, when one study was removed (study conducted by Ikeda et al.), a significant association was determined for the heterozygous (OR=1.27, 95% CI: 1.02-1.57, p<0.031) and dominant (OR=1.24, 95% CI: 1.01-1.52, p<0.041) genetic models (see supplement data). This posits that, for non-Asians, the L55M polymorphism increases the risk of developing AMD (Table 2). However, for the Asian population, the heterozygous, dominant, and allelic genetic models demonstrated a benefit/protective factor. When the cohort was stratified into exudative and non-exudative cases, there were no associations observed (Table 3).

**Table 2:** Effect of PON1 polymorphisms on the development of AMD by region

**Region**	**Genetic model**	**N^a^**	Association for the L55M polymorphism^c^				Association for the Q192R polymorphism^c^			
			OR	95% CI	p-value	Model^b^	OR	95% CI	p-value	Model^b^
Non-Asian	Heterozygous	4	1.27	1.02-1.57	0.031^*^	Fixed	1.06	0.85-1.33	0.583	Fixed
	Homozygous	4	1.07	0.54-2.10	0.850	Random	0.95	0.26-3.39	0.931	Random
	Dominant	4	1.24	1.01-1.52	0.041^*^	Fixed	1.04	0.65-1.65	0.885	Random
	Recessive	4	0.97	0.56-1.67	0.910	Random	0.51	0.14-1.81	0.297	Random
	Allelic	5	1.11	0.96-1.28	0.159	Fixed	0.94	0.65-1.35	0.725	Random
Asian	Heterozygous	1	0.29	0.11-0.79	0.016^*^	Fixed	1.07	0.38-3.00	0.894	Fixed
	Homozygous	1	0.41	0.05-3.74	0.428	Fixed	2.20	0.79-6.11	0.131	Fixed
	Dominant	1	0.31	0.12-0.77	0.012^*^	Fixed	1.52	0.57-4.04	0.401	Fixed
	Recessive	1	0.48	0.05-4.37	0.514	Fixed	2.08	1.16-3.70	0.013^*^	Fixed
	Allelic	1	0.35	0.15-0.80	0.013^*^	Fixed	1.63	1.06-2.53	0.028^*^	Fixed

AMD= age-related macular degeneration; OR= odds ratio; PON1= paraoxonase-1; 95% CI= 95% confidence interval.

a = N= number of studies included in the analysis.

^b^= Depending on the level of heterogeneity, either the random or fixed effects model was used.

c = Pooled effects were calculated using the Comprehensive Meta-Analysis software Version 2.2.

* = p-values <0.05 (two-tailed) were considered significant.

**Table 3:** Effect of PON1 polymorphisms on the development of AMD by pathology

Pathology	Genetic model	N^a^	Association^b^			Comparison
			OR	95% CI	p-value^c^	p-value^d^
*L55M* Exudative	Heterozygous	4	0.97	0.57-1.65	0.921	0.856
	Homozygous	4	0.74	0.28-1.98	0.551	0.627
	Dominant	4	0.89	0.50-1.59	0.701	0.936
	Recessive	4	0.71	0.31-1.64	0.425	0.454
	Allelic	4	0.83	0.51-1.36	0.461	0.419
Non-exudative	Heterozygous	3	0.88	0.58-1.34	0.563	N/A
	Homozygous	3	0.98	0.52-1.83	0.948	N/A
	Dominant	3	0.89	0.60-1.32	0.560	N/A
	Recessive	3	1.07	0.60-1.93	0.815	N/A
	Allelic	3	0.95	0.71-1.27	0.724	N/A
*Q192R* Exudative	Heterozygous	4	1.10	0.86-1.41	0.432	0.848
	Homozygous	4	0.96	0.23-3.97	0.957	0.956
	Dominant	4	1.15	0.68-1.94	0.609	0.783
	Recessive	4	0.85	0.26-2.86	0.799	0.993
	Allelic	4	1.07	0.63-1.84	0.801	0.354
Non-exudative	Heterozygous	3	1.16	0.75-1.81	0.512	N/A
	Homozygous	3	0.91	0.30-2.79	0.874	N/A
	Dominant	3	1.04	0.68-1.59	0.851	N/A
	Recessive	3	0.85	0.30-2.39	0.755	N/A
	Allelic	3	0.97	0.62-1.54	0.910	N/A

AMD= age-related macular degeneration; N/A= not applicable; OR= odds ratio; PON1= paraoxonase-1; 95% CI= 95% confidence interval.

a = Number of studies included in the analysis.

b = Pooled effects were calculated using the Comprehensive Meta-Analysis software Version 2.2.

c = p-values <0.05 (two-tailed) indicate a significant association between the polymorphism and type of AMD.

d = p-values <0.05 (two-tailed) indicate a significant difference between the two pathologies.

### PON1 Q192R polymorphism increases the risk of developing AMD in Asian populations

**Figure 3:**
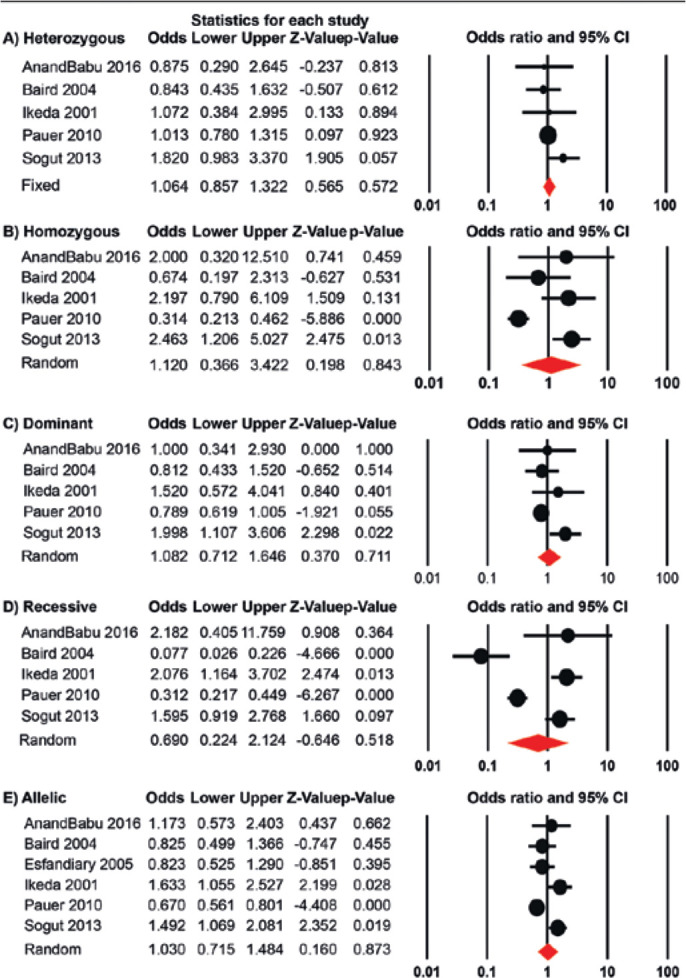
Forest plots to determine the risk of developing age-related macular degeneration (AMD) associated with the paraoxonase-1 (PON1) Q192R polymorphism for the heterozygous (A), homozygous (B), dominant (C), recessive (D), and allelic (E) genetic models. The circles and horizontal lines correspond to the study-specific crude odds ratio (OR) and 95% confidence interval (95% CI), respectively. The area of the circles reflects the study-specific weight. The diamond represents the pooled OR and 95% CI, determined using either the fixed or random effects model, depending on the level of heterogeneity. Plots were generated using the Comprehensive Meta-Analysis software Version 2.2.

Only the heterozygous genetic model did not present significant heterogeneity; thus, it was analyzed using the fixed effects model. However, none of the genetic models demonstrated a significant association between the Q192R polymorphism and AMD (Figure 3). When one study was removed and the pooled OR was re-calculated, only the results of the homozygous genetic model were sensitive to the study conducted by Pauer et al.^(16)^, which demonstrated a positive association (OR=1.86, 95% CI: 1.07-3.21, p=0.027) (see Supplemental data).

When the cohort was grouped by ethnicity, a significant association was detected in the Asian population for the recessive and allelic genetic models (Table 2). This posits that, for Asians, the Q192R polymorphism increases the risk of developing AMD. However, for non-Asian population, there was no association observed. When the cohort was stratified into exudative and non-exudative cases, there were no associations observed (Table 3).

### Heterogeneity, publication bias, and small study effects

**Figure 4:**
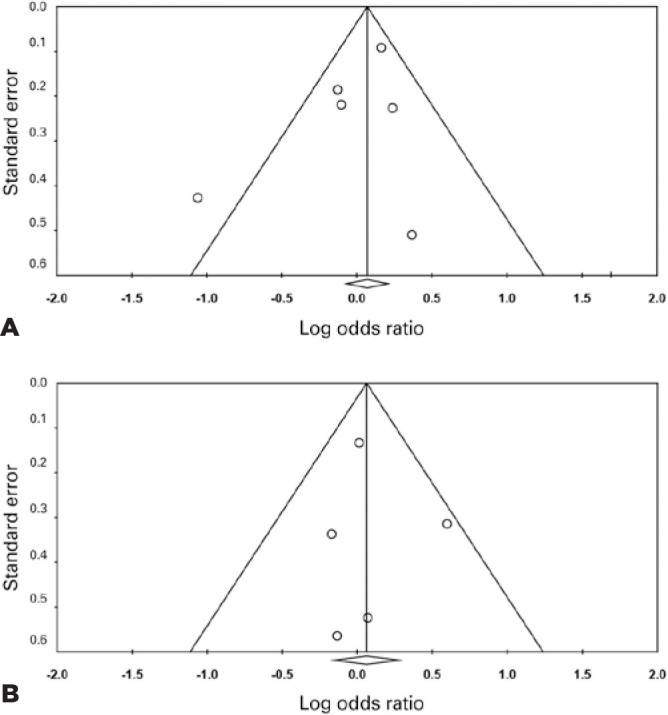
Begg’s funnel plot for publication bias. (A) For the paraoxonase-1 (PON1) L55M polymorphism, there was no detrimental asymmetry observed (allelic genetic model). (B) Similarly, there was no detrimental asymmetry observed for the PON1 Q192R polymorphism (heterozygous genetic model). Each point represents a separate study. For all Begg’s funnel plots, see supplemental data.

Sources of heterogeneity were assessed by examining Galbraith plots (Supplemental information). For the L55M polymorphism, consistently across the five genetic models, only the study performed by Ikeda et al.^(11)^ (the Asian study) was indicated as the most likely cause of heterogeneity. However, for the Q192R polymorphism, the studies conducted by Ikeda et al.^(11)^, Pauer et al.^(16)^, and Söğüt et al.^(12)^ were indicated as sources of heterogeneity; however, there was no common characteristic identified among these studies. We examined the funnel plot for each polymorphism to determine publication bias and small study effects. The funnel plot did not demonstrate publication bias (Figure 4). Moreover, there was no bias or a correlation observed, as determined by Egger’s test or the Begg-Mazumdar test, respectively.

## DISCUSSION

An increase in lipid peroxidation is associated with progression of AMD^(4)^; therefore, the main anti-oxidant enzyme carried by high-density lipoprotein particles, PON1’s enzymatic activity or polymorphisms that affect its activity could augment the risk of developing AMD^(17)^. Indeed, we demonstrated that the L55M polymorphism increased the risk of developing AMD for non-Asians. However, for the Q192R polymorphism, only Asians presented with an increased risk.

As expected, most models presented significant heterogeneity. Although some studies identified AMD using the Age-related Eye Disease Study (AREDS) criteria, Pauer et al.^(16)^ sub-categorized their cohort into one of four groups using the criteria defined by the AREDS, in which only the fourth category could be classified into exudative and non-exudative cases. The effect these polymorphisms have on AMD stage development is poorly understood and could lead to a source of heterogeneity. As illustrated in the study conducted by Pauer et al.^(16)^, the Category 1 group did not demonstrate difference in allelic or genotypic distribution compared with the control group for either polymorphism; however, Categories 2-4 presented a significant risk, as observed with the Q192R polymorphism^(16)^. Thus, it is possible that the sub-categorical distributions of each of the remaining studies included in this meta-analysis could vary, increasing the heterogeneity. Another source of heterogeneity could be related to the subject’s obesity and dietary habits, as well as environmental factors. Few studies have shown that a high diet in polyunsaturated fatty acids, zinc or copper, and polyphenols or carotenoids, while avoiding red meat, decreases the risk of developing AMD^(7)^. Owing to the geographic locations of the included studies, it is reasonable to assume that the diets differ between each study. Smoking^(7,18)^ and alcohol consumption^(8)^ were shown to decrease PON1 activity, augmenting the risk of developing AMD. Moreover, the quality (wine versus beer) and quantity (social versus binge) of alcohol consumption can lead to a benefit against developing AMD or increase its risk, respectively^(8)^. In this analysis, none of the studies took into consideration the smoking status or alcohol consumption. None of the studies corrected their data based on nutritional intake or obesity. Lastly, the age and sex of the patients could also lead to sources of heterogeneity; studies showed that older subjects^(19,20)^ and males^(19)^ are more prone to developing AMD.

The L55M polymorphism has been shown to affect serum concentrations and subsequently overall enzymatic activity^(21)^. It was expected that this polymorphism would increase the risk of developing AMD. Indeed, only for non-Asians, models consisting of the heterozygous genotype presented a significant risk of AMD development. This suggests that an over-dominant pattern exists for non-Asians. Nevertheless, for the Asian population, a possible protective factor was observed; nevertheless, this result is based on one study and replicative studies are warranted. The mechanism for this difference remains elusive, but could be associated with diet and other lifestyle factors.

For the Q192R polymorphism, overall, there was no association with the development of AMD. However, there was an increased risk in the Asian population; yet again, this observation was based on one study and replicative studies are required. The Q192R polymorphism is located in the region for substrate identification. A study conducted by Aviram et al. demonstrated that, when the substrate is paraoxon, PON1 more rapidly hydrolyzes it with the R-isoform, whereas diazoxon is hydrolyzed by the Q-isoform^(22)^. Thus, the effect of the Q192R polymorphism in Asians may be explained by diet. In support of this, the Q192R polymorphism Q-isoform is more efficient in inhibiting the oxidation of low-density lipoprotein compared with the R-isoform. Moreover, the Q-isoform is most prevalent in non-Asian populations and their descents^(23)^, whereas the R-isoform predominates in Asian populations^(24)^.

Although PON1 activity is dependent on two calcium ions. One calcium ion is associated with the enzyme structure, while the other is linked to enzymatic activity with respect to substrate positioning. According to Laird et al., mercury and selenium positively affect PON1 activity, whereas cadmium decreases this activity^(25)^. However, Ginsberg et al. reviewed the effect of metal ions on PON1 activity, indicating that barium, zinc, copper, lead, mercury, cobalt, cadmium, and nickel inhibit PON1 activity *in vitro*^(26)^. Nevertheless, exposure to metals through either dietary intake (e.g., methylmercury from fish)^(27)^ or lead from environmental exposure^(28)^ significantly decreases PON1 activity. In Asians, cadmium, lead, and mercury serum concentrations were significantly higher than those measured in all other non-Asian populations studied^(29)^; this difference is mostly attributed to a diet of fish and/or rice^(30)^. Thus, any effect by PON1 and its polymorphisms on disease development should take into consideration diet with respect to metal concentration, PON1 polymorphism haplotypes, and any disease-specific substrate of PON1. These three factors, individually or in any possible grouping, could be the reason for the differential effects determined in this study.

This study had a few limitations. Firstly, the results are presented as un-adjusted ORs. As mentioned above, the association of environmental factors (e.g., smoking, dietary intake, and lifestyle) could affect the risk of developing AMD. Therefore, future studies should consider these factors. Secondly, only articles published in English were selected. Latin American and Asian countries that publish articles in Chinese, Spanish or Portuguese, were not included and may have affected coverage. Thirdly, small study effects are most likely present. Although our study analyzed four non-significant studies showing a significant effect, the low number of studies may not offer sufficient power to detect an association; thus, the results must be assessed cautiously. Additional studies with larger sample sizes and containing more detailed information are warranted.

In conclusion, we have determined that the PON1 L55M polymorphism increases the risk of developing AMD in non-Asian populations, whereas in Asian populations, this polymorphism exerts a protective effect. However, for the PON1 Q192R polymorphism, only the Asian population demonstrated a risk of developing AMD.
